# Prognostic Significance of New Immunohistochemical Markers in Refractory Classical Hodgkin Lymphoma: A Study of 59 Cases

**DOI:** 10.1371/journal.pone.0006341

**Published:** 2009-07-22

**Authors:** Danielle Canioni, Bénédicte Deau-Fischer, Pierre Taupin, Vincent Ribrag, Richard Delarue, Jacques Bosq, Marie-Thérèse Rubio, Damien Roux, Viorel Vasiliu, Bruno Varet, Nicole Brousse, Olivier Hermine

**Affiliations:** 1 Department of Pathology, Necker Hospital, Assistance Publique-Hôpitaux de Paris, University Paris-Descartes, Faculté de Médecine, Paris, France; 2 Department of Hematology, Necker Hospital, Assistance Publique-Hôpitaux de Paris, University Paris-Descartes, Faculté de Médecine, Paris, France; 3 Department of Biostatistics, Necker Hospital, Assistance Publique-Hôpitaux de Paris, University Paris-Descartes, Faculté de Médecine, Paris, France; 4 Department of Hematology, Gustave Roussy Institute, Villejuif, France; 5 Department of Pathology, Gustave Roussy Institute, Villejuif, France; Faculdade de Medicina, Universidade de São Paulo, Brazil

## Abstract

Although most classical Hodgkin lymphoma patients are cured, a significant minority fail after primary therapy and may die as result of their disease. To date, there is no consensus on biological markers that add value to usual parameters (which comprise the International Prognostic Score) used at diagnosis to predict outcome. We evaluated 59 patients (18 with primary refractory or early relapse disease and 41 responders) for bcl2, Ki67, CD20, TiA1 and c-kit expression by semi-quantitative immunohistochemical study and correlated the results with the response to treatment.

The results showed that expression of bcl2 and CD20 in Hodgkin and Reed Sternberg cells, and expression of TiA1 in micro-environmental lymphocytes, and c-kit positive mast cells in microenvironment, were independent prognostic markers. These novel cHL markers could be used in association with clinical parameters to identify newly diagnosed patients with favorable or unfavorable prognosis and to better tailor treatment for different risk groups.

## Introduction

Classical Hodgkin lymphoma (cHL) is a highly curable lymphoma and about 80% of patients can be cured with modern treatment strategies [1], [2]. In spite of great clinical progress, a significant minority of cHL experiences treatment failure after primary chemotherapy including a first line of anthracyclin-based regimen [2], [5]. Patients with refractory cHL represent 5 to 10% of cHL. Many of these patients have a poor overall survival of 26% at 5 years [6]. A better biological characterization of such primary refractory patients might allow the use of targeted therapeutic strategies earlier during the course of the disease [1], [7]. Most prognostic score systems used to date for advanced stage of the disease, including the International Prognostic Score (IPS), which incorporates seven clinical and laboratory parameters, failed to accurately identify patients with unfavorable responses to therapy [1], [7]–[9] . Therefore, current attempts to identify high risk patients who may benefit of novel therapies have not proven to be successful to date [10]–[13]. Several markers such as serum levels of soluble CD30 [1], [14] and some interleukins [15], [16] might provide additional prognostic information to the clinical models. Different studies reported a correlation between markers of cell activation and/or differentiation, cell cycle and apoptosis deregulation, Epstein Barr Virus (EBV) detection in the neoplastic Hodgkin and Reed Sternberg (H/RS) cells and the clinical outcome of cHL patients [17], [18]. A peculiar feature of Hodgkin disease is that neoplastic cells constitute less than 1% of the cellular population of HL-involved tissues since H/RS cells are interspersed among a heterogeneous population of non malignant reactive cells [19]. Several studies have documented that H/RS cells are highly interactive with this microenvironment through direct cell contacts and production of various cytokines and chemokines [14], [16], [20].

To further evaluate the prognostic significance of new biological markers in cHL, we compared the expression of bcl2, Ki67 and CD20 expression in H/RS cells of refractory and early relapse patients to that of responder patients. In addition, we compared the expression of TiA1 in surrounding T lymphocytes as a putative marker of an anti-tumoral immune response [21]–[23] in both groups of patients. We also looked at the expression of c-kit to evaluate the presence of mastocytes, which might modify the behaviour of cHL [24], [25]. These results were analyzed statistically in conjonction with clinical and laboratory parameters and were correlated with treatment response.

## Materials and Methods

### Patients

A total of 65 patients were retrospectively collected from 1997 to 2004 in 2 hematology centres (Necker Hospital and Gustave Roussy Institute, Paris France): all available poor prognosis patients were first identified (18 patients with primary refractory disease or early relapse) and the control group (47 responders) was randomly selected. Patients were eligible for this study if they fulfilled the following criteria: (1) diagnosis based on a lymph node biopsy (or another organ) performed before any treatment; (2) paraffin-embedded formalin-fixed tissue blocks from the diagnosis lymph node (or another organ) available for immunohistochemical studies; (3) a minimum follow-up of 2 years and (4) a negative human immunodeficiency virus (HIV) serology. Our clinical trial has been performed after having been approved by the authors' institutional review board of the 2 hospitals involved in this study. The data of patients were analyzed anonymously and all clinical investigation has been conducted according to the principles expressed in the Declaration of Helsinki.

Patients received conventional chemotherapy-based treatments [(MOPP (mechlorethamine, vincristine, procarbazine, and prednisone), ABVD (doxorubicine, bleomycine, vinblastine, and dacarbazine) or the combination of both or BEACOPP (bleomycine, etoposide, doxorubicine, cyclophosphamide, vincristine, procarbazine, prednisone)] and radiotherapy in stages I and II. Treatment decisions were not based on molecular and/or immunohistochemical features.

Records of patients were reviewed by two hematologists (BD and VR). Clinical, analytical, therapeutic and follow-up data were collected in a data base including age, sex, Ann Arbor staging, B symptoms (weight loss, fever, drenching sweat), histologic type, number of lymph nodes and extranodal sites, erythrocyte sedimentation rate, serum level of lactate deshydrogenase, albumin, β2-microglobulin, hemoglobin concentration, lymphocytopenia, response to therapy (complete response versus treatment failure), overall survival from time of diagnosis (or beginning of treatment), and for responding patients, relapse free survival from the end of therapy. Complete remission was defined as the resolution of clinical and radiological evidence of disease for a minimum of 4 weeks. Primary refractory disease was defined as an absence of treatment response or the evidence of disease progression within the first 3 months period after the end of treatment. Early relapse was defined as disease progression in the first year after diagnosis. Patients were considered as responders when they remained in complete response for at least one year after diagnosis. All data were checked for validity and coherence. Errors and missing values were reported to the contributors and investigators for correction or confirmation.

Six cases were excluded because of lack of tissue samples in three of them and unsatisfactory immunohistochemical staining in 3 other cases. Thus, 59 out of the 65 cases collected were considered eligible for the final statistical analysis: 18 patients with primary refractory disease or early relapse and 41 responders.

### Pathological and immunohistochemical procedures

All cases were reviewed independently by one hematopathologist (DC) who was blinded as to treatment response. Diagnosis confirmation of cHL and histological subclassification were performed on slides stained with hematoxylin and eosin, Giemsa and Gordon-Sweet stains, following the Rye classification in four main types: lymphocyte predominance, nodular sclerosis, mixed cellularity and lymphocyte depletion. Priority in the diagnosis was conferred to the morphological study.

Tissue samples were fixed in 10% formalin and embedded in paraffin. Most of them were lymph nodes, 3 were extra nodal specimens in the absence of adenopathy (liver, spleen and bone tissue). Immunohistochemistry was performed using automated immunohistochemical stainers (Techmate 500 Dako, Dako Cytomation). Tissue sections were incubated with the following antibodies: CD20/L26, CD3/F7.2.38, Ki67/MiB1, bcl2/124 (all from Dako Cytomation), c-kit/CD117, and TiA1/2G9 (from Immunotech). All of them are mouse monoclonal antibodies except c-kit, which is a rabbit polyclonal antibody. After incubation, immunodetection was performed with the ChemMate Dako EnVision detection kit (Dako Cytomation) employing Peroxidase/Diaminobenzidine. Other cells in the sections served as internal controls for each antibody.

**Figure 1:**
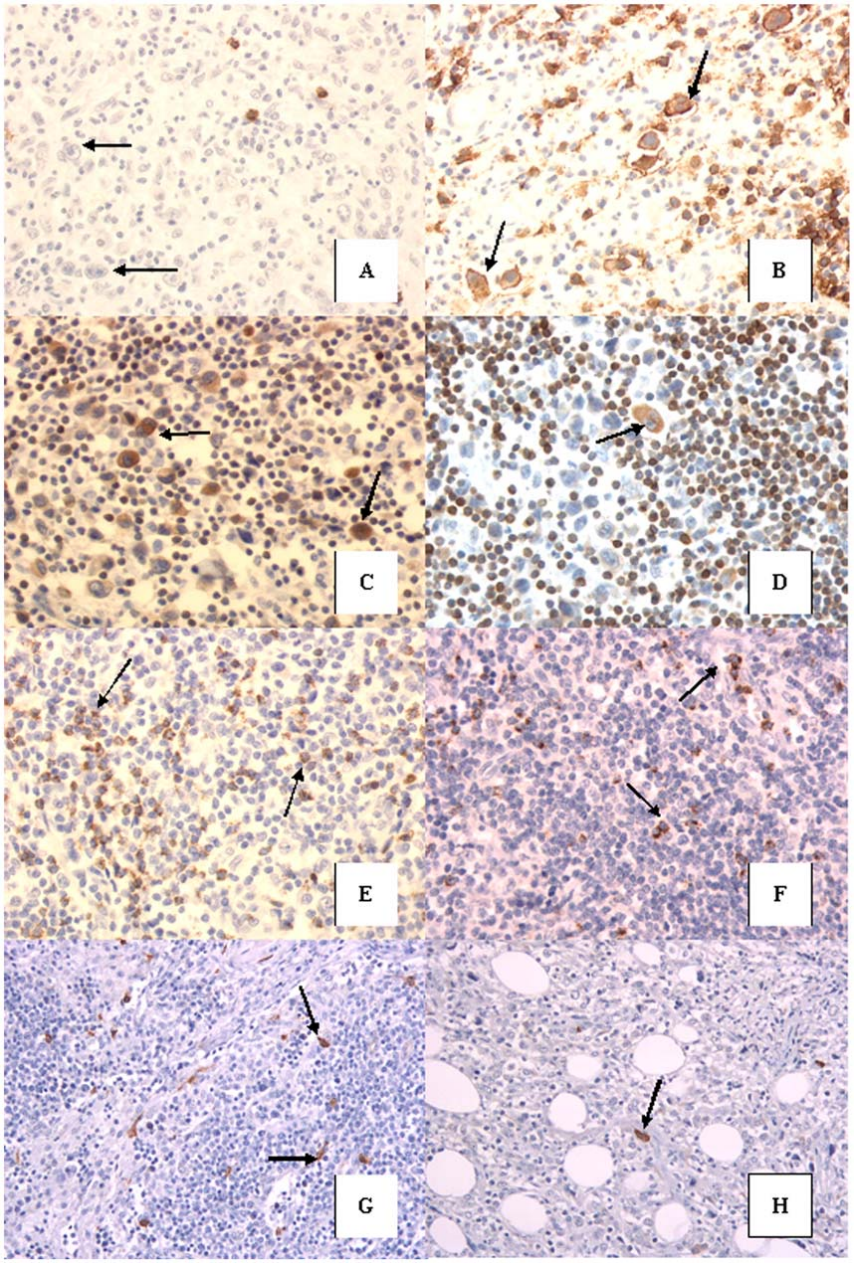
**A & B**: Rare or no (arrows) H/RS cells stained with CD20 in refractory cHL (**A**) compared to the overexpression of CD20 on H/RS cells in a responder cHL (arrows) (**B**) (original magnification ×400). **C & D**: Numerous H/RS cells stained with bcl2 in a refractory cHL (arrows)(**C**) comparing to rare cells in a responder case (arrow)(**D**) (original magnification ×400). **E & F**: TiA1 expressed on numerous small lymphocytes in a refractory cHL (arrows) (**E**) and less frequent cells stained with this antibody in a responder cHL (arrows) (**F**) (original magnification ×400). **G & H**: c-kit stained frequent mast cells in a refractory cHL (arrows) (**G**) compared to rare mast cells expressing c-kit in a responder cHL (arrows) (**H**) (original magnification ×400).

Analysis of sections was assessed using a semi-quantitative method which differed depending on the antibody used. For CD20 and CD3 antibodies, the number of negative or positive H/RS cells was evaluated. In positive cases defined by the presence of at least 10% of antigen expressing cells, the number of stained H/RS cells among 200 randomly selected H/RS cells was assessed. For Ki67 and bcl2 antibodies the number of stained H/RS cells on 200 randomly selected H/RS cells was evaluated. The number of lymphocytes stained with TiA1 antibody was assessed on 10 fields randomly selected per high power field (hpf) (×400). For c-kit antibody, the number of mast cells was evaluated on hpf (×400) following the same process than that with TiA1 antibody (Figure 1).

### Statistical analyses

Patient characteristics were compared using the Fisher exact test for categorical variables and the Wilcoxon test for continuous variables in order to identify factors that might significantly influence survival and initial response to treatment. Only cases with all of the required data were selected for statistical analysis. The analysis was thus performed in 59 patients from the initial group of 65 patients. For this initial analysis these tests were used to compare the means of the continuous variables between patients. Results were expressed as median (percentile 25–75% except for CD20 since in this case the exact number of positive cells has not been determined when it was under 10%). For these results to be applied to individual cases, a specific analysis based on cut-off levels has been required.

An univariate analysis was performed for each clinical variable and for the tumor expression in tissue of the five analyzed markers (Ki67, CD20, bcl-2, c-kit and TiA1), first with continuous variable and then with binary variable.

A multivariate analysis was performed to identify the factors that might be of independent significance in influencing overall survival and failure to achieve complete response. Variables included were: age (over or under 45 years), Ann Arbor stage (I–II, versus III–IV), B symptoms (present or absent), gender, number of lymph nodes involved (over or under 3), erythrocyte sedimentation rate (over or under 30), albumin (over or under 40 gr/l), hemoglobin rate (over or under 10.5 g/dl), LDH (>1N or N), lymphocytes (over or under 600), CD20, bcl-2, TiA1 and c-kit (with continuous variable and then binary variables). Immunohistochemical markers were included in a logistic-regression model, to provide adjusted tests of the relationship between each immunohistochemical marker and the refractory status.

All tests were two-sided and p-values of 0.05 or less were considered as statistically significant. Statistical analyses were conducted with the R software, version 2.0.

## Results

### Clinical and usual Biological findings

A total of 59 patients randomly selected in the 2 centres formed the basis of the study distributed among 18 refractory or early relapse cHL and 41 responders to treatment. Their clinical features and the results of the univariate analysis are summarized in Table 1.

**Table 1:** Characteristics of patients & IHC markers (univariate analysis).

	Number of pts n = 59	Primary Refractory or early relapse n = 18	Responders n = 41	Wilcoxon *p*
**Sex**				
Female	28	11	17	
Male	31	7	24	0.2
**Age, years**				
<45 y	44	16	28	
>or = 45 y	15	2	13	0.1
**Stage**				
I–II	44	12	32	
III–IV	15	6	9	0.5
**Histological Sbtype**				
Nodular sclerosis	54	17	37	
Others	5	1	4	
**B symptoms**				
No	31	5	26	
Yes	25	13	15	0.02
**Sedimentation Rate**				
>30	37	16	21	
<or = 30	22	2	20	0.008
**Albumin**				
>or = 40	41	11	30	
<40	18	7	11	0.7
**Hemoglobin level**				
>or = 10.5	45	13	32	
<10.5	14	5	9	0.4
**LDH level**				
<or N	46	9	37	
>N	13	9	4	0.001
**Lymphocytes**				
>or = 600	53	14	39	
<600	6	4	2	0.08
**First Line Therapy**				
ABVD	47	14	33	
EBVP	8	3	5	
BEACOPP	3	1	2	
**IHC markers C.V: median (range 25–75%)**				
% HRS CD20+		15	34	0.03
% HRS bcl2 high		51 (5.75–75.75)	12 (7.5–27.5)	0.01
Number of L TiA1+		42 (25.75–50.75)	21 (14–30)	0.0005
Number of MC ckit+		9 (5–13.5)	3.8 (2.4–6)	0.001
**Binary variable: nber of patients(%)**				
HRS CD20>30%		2 (11%)	17 (41%)	0.03
HRS bcl2>40%		12 (66%)	8 (19%)	0.0008
L TiA1+>30		12 (66%)	12 (29%)	0.01
Mast cells>6		12 (66%)	12 (29%)	0.01

**Legends**: pts = patients sbtype = subtype L = lymphocytes MC = mast cells nber = number IHC:immunohistochemical CV: continuous variable HRS: Hodgkin and Reed Sternberg cells.

Initial diagnosis of cHL was confirmed by histological analysis of a lymph node in 56 cases or an extra nodal site in 3 cases. Most patients presented a nodular sclerosis subtype of cHL regardless whether they were refractory (17/18cases, 94.5%) or responders (37/41cases, 90%). The other histological type was mixed cellularity for 5 cases.

In univariate analysis the group of responder patients and that of those with early relapse or primary refractory disease were comparable according to the following characteristics: age, sex, histopathological type, stage and number of involved lymph nodes, albumin, hemoglobin concentration and lymphocytes. However, patients with refractory or early relapse cHL showed significantly more B symptoms (*p = 0.02*), higher sedimentation rate (*p = 0.008*) and LDH levels (*p = 0.001*).

### Tumoral cells characteristics

The expression of CD20 in Hodgkin or Reed-Sternberg (H/RS) cells was heterogeneous and significantly less frequent in refractory or early relapse cHL (15% of HRS positive cells) than in responders (34% of HRS positive cells) (*p* = 0.03) (Figure 1A). Thus, CD20 seems to be overexpressed on H/RS cells of responding patients. The median value of bcl2 positive H/RS represented 51% of HRS cells in refractory or early relapse patients and only 12% of H/RS cells of responders. This difference was statistically significant (*p = 0.01*). (Figure 1B).

Numerous small lymphocytes were stained with CD3 (no difference between both groups) but this antibody was negative on H/RS cells in our series (data not shown). No difference was found between Ki67 expression in responders and in refractory cHL since H/RS cells were diffusely stained with Ki67 in both groups (>80%).

### Non neoplastic tumor-infiltrating cells characteristics

Refractory or early relapse cHL had significantly higher numbers of infiltrating cytotoxic TiA1+ small lymphocytes (median = 42/hpf) than chemosensitive ones (median = 21/hpf) (*p = 0.0005*) (Figure1C). TiA1 positive cells were scattered in the lymphoma or in foci. C-kit antibody (CD117) was negative on H/RS cells but allowed to evaluate the number of mast cells stained, which was significantly higher in refractory cHL (median = 9/hpf) than in responders (median = 3.8/hpf)(*p = 0.001*) (Figure 1D). Thus, TiA1+ lymphocytes and mast cells are more numerous in the non tumoral background of refractory or early relapse cHL than in responding patients.

In summary, in the univariate statistical analysis immunohistochemical markers including CD20, bcl2, TiA1 and c-kit were significantly different in refractory or early relapse and responders patients and were correlated with the treatment response.

### Multivariate statistical analysis

Multivariate statistical analysis including bcl2, TiA1, c-kit and CD20 markers (with continuous variable) revealed that only low CD20 (*p = 0.04*), (or<30% with binary variable (*p = 0.04*)), high bcl2 (*p = 0.01*) (>40% with binary variable (*p = 0.008*)), and high TiA1 (*p = 0.003*) (>30% with binary variable (*p = 0.002*)) expression remained independently associated with the refractory status of cHL (Table 2).

**Table 2:** Multivariate analysis of immunohistochemical markers.

	Hazard Ratio	95% Confidential Interval	*p*
**Continuous variable**			
CD20			0.04
Bcl2			0.01
TiA1			0.003
c-Kit			0.4
**Binary Variable**			
CD20	7.55	0.99–57.5	0.04
Bcl2	8.58	1.63–45.1	0.008
TiA1	5.99	1.21–29.7	0.002
c-Kit	2.89	0.61–13.7	0.17

We also analyzed relationships between molecular markers and clinical and common biological parameters. A significant link was found between the number of mast cells assessed by ckit expression and B symptoms in the refractory cHL (*p = 0.017*). This link was not present in the responders (*p = 0.76*) (data not shown).

## Discussion

This retropective study of 59 patients presenting either refractory and early relapse or responding classical Hodgkin lymphoma provides evidence that new immunohistochemical markers taking into account apoptosis markers of tumoral cells as well as surrounding microenvironment could be predictive of response to treatment in cHL patients.

Some markers based on the presumed pathogenesis of cHL have been reported as having a prognostic value for patients with cHL but there is no consensus on biologic markers that add prognostic value to the clinical parameters [1], [7], [18], [21], [22]. Moreover, none of them was studied on refractory patients compared to responding patients.

The pathogenesis of cHL remains poorly understood and single-cell techniques have proven that H/RS cells are derived from germinal center B cells [26], [27] but lack immunoglobulin expression. However, although normal germinal center B cells that lack functional high affinity antibody undergo apoptosis within germinal center, H/RS cells may escape this programmed cell death [28], [29]. Thus alteration of apoptosis regulators expression in H/RS cells [7], [30] may prevent apoptosis caused by the absence of the functional B-cell receptor and may explain resistance to treatment [31], [32]. To test this hypothesis we have quantified the percentage of H/RS cells that expressed the anti-apoptotic bcl2 protein. We found that bcl2 was highly expressed in HRS cells of refractory or early relapse cHL and was an independent factor in multivariate analysis (*p = 0.01*). This finding extends in the refractory population the results recently reported by Smolewski & al and Garcia & al that showed a shorter survival in patients with high expression of bcl2 [17].

Although H/RS cells originate from germinal center B cells, the classical B-cell marker CD20 is expressed in only 20–30% of cHL [27], [33]. The prognostic significance of CD20 expression in cHL is controversial and a matter of ongoing debate [33]–[37]. Indeed, some studies found no significant association between CD20 expression in H/RS cells and the outcome of cHL [34], [36], whereas others reported a worse clinical outcome in CD20-positive patients [35], [37]. Surprisingly, we found that the lack of CD20 expression was associated with refractory disease and remained significant in multivariate analysis. Our results on refractory cHL are close to those of Tzankov & al [33] who reported a significantly higher frequency of disease relapses in the CD20 negative group compared to the CD20 positive cHL with a follow up of 12 years and a cut off of 10% of cells expressing this antigen. The reason for a significant higher CD20 expression in H/RS cells in responders compared to that of refractory cHL is unknown. CD20 resembles a Ca2+ ion channel and is involved in signal transduction for B-cell differentiation and proliferation. Tzankov [33] suggested that an increase of Ca2+ permeability in H/RS cells along with chemotherapy and/or radiotherapy could decrease apoptotic resistance or even activate programmed cell death, as it does in other lymphomas [38]. It is also possible that CD20 expression could be a marker of a different gene expression program associated with a better response to treatment and then with a better outcome [28], [29]. Moreover it raises the question of whether there is a correlation between this expression in H/RS cells and the response to anti-CD20 therapy in cHL [34], [39]. Indeed, recent studies of non-Hodgkin lymphomas demonstrated that the quantitative expression of CD20 molecules on distinct histological types of lymphoma correlates with rituximab efficacy [39], [40].

High proliferation index (Ki67) is described as an adverse prognostic factor in non HL. Morente & al [41] reported the same results in a study of 140 patients with HL and showed that a high proliferation index influenced lower overall survival and failure to achieve complete remission. In our series, however, there was no significant difference of proliferation index between responder cHL and refractory patients since Ki67 was highly expressed in both groups (data not shown).

Hodgkin lymphoma is characterized by a minority of tumour cells surrounded by a large amount of non-neoplastic cells [19]. Knowledge about the biology of the disease particularly the interaction between H/RS cells and the surrounding cells is essential in order to improve its diagnosis and treatment. Although the composition of the infiltrate of the background is heterogeneous, most cells are CD4+ lymphocytes. Recent studies of HL have shown that infiltrating lymphocytes in cHL lymph nodes are predominantly CD4+, CD25+ regulatory T cells which have suppressive functions and induce a profoundly immunosuppressive environment [21]–[23]. This finding suggests that the presence of regulatory T cells in the reactive background may explain the inhibition of the antitumoral host immune response observed in cHL [23]. Our study aimed at assessing the relevance of cytotoxic T cells (CTL) present in the background of cHL samples in the response to treatment. We found that a high proportion of TiA1+ cells in the infiltrate represents a prognostic factor that negatively influenced the response to treatment. Indeed, patients who were refractory to treatment showed a significant higher count of TiA1+ cells in the samples studied compared to responding cHL (*p = 0.0005*). Furthermore, this marker remains an independant factor predictive of a poor response to treatment in a multivariate analysis (*p = 0.003*). This result is similar to the study of Lejeune et al who reported more TiA1+ cells (and a lower proportion of FOXP3+ cells) in the samples of patients who relapsed compared to the features at the time of diagnosis [21], [22]. This finding suggests that primary refractory cHL shows the same pattern of anti-tumoral host immune response as the relapsing one. It could seem inappropriate that a lot of CTL in the background of cHL be associated with a worse response to treatment of these patients. The hypothesis for explaining these results could be the consequence of defective CTL against H/RS cells that may paradoxically provide survival factors. It remains however to be determined by which mechanisms H/RS or other infiltrating cells inhibit CTL function.

Among the reactive cells which surround H/RS cells, mast cells are important since they are CD30ligand (L)-positive and they activate H/RS cells in vitro through CD30-CD30L interaction but also through soluble factors like interleukin 8, 9 or 13 [24], [25], [42]. Mast cells are the predominant CD30L-positive cells and play a role in many inflammatory disorders and have important immunoregulatory functions through cytokines and accessory molecules expression [24]. They are often present in tumors but their role in cHL has rarely been explored [25], [43]. As reported by Molin & al [43], we found that a high number of mast cells expressing c-kit receptor had a clinical importance in the response to treatment. Indeed, for Molin & al [43], significant mast cell infiltration was associated with a worse relapse-free survival and in our series it was much more frequent in refractory or early relapse cHL than in responding patients (*p = 0.001*). The CD30L present on mast cells is functionnally active and can provide stimulatory signals to H/RS cells [24]. Thus, mast cells that constitute an important portion of the infiltrating reactive cells may contribute to tumor progression in cHL. Mast cells might be specifically attracted to the tumor area by chemokines produced by H/RS cells and studies revealed several chemokines in HL lines among which CCL5 (RANTES) [24], [43], [44]. This chemokine could be a good candidate responsible to mast cell attraction into tumor tissue in cHL. Knowing that mast cells express c-kit receptor and that, during our experiment they were significantly more numerous in refractory cHL than in responders, drugs inhibiting the tyrosine kinase activity of ckit, like Imatinib (Gleevec), in combination with chemotherapy might have therapeutic value in refractory c-HL (45). Beside this effect on cHL prognosis the presence of mast cells is correlated with B symptoms. This finding may be related to cytokines and mediators release by activated mast cells [45], [46]. To confirm this hypothesis, in future prospective studies it would be interesting to assess the value of histamine and tryptase levels and whether or not they are correlated with some systemic symptoms including pruritus, night sweats and/or weight loss.

In summary, this retrospective study of 59 patients presenting either a refractory and early relapse cHL (18 cases) or a responding disease (41 cases), provides evidence that we can reasonably propose new immunohistochemical markers to predict the response to treatment of cHL based both on features of tumoral cells but also on microenvironment. Our immunohistochemical study reveals that in refractory cHL, H/RS cells present at diagnosis an overexpression of bcl2 marker and a frequent absence of CD20 expression and that there was an excess of cytotoxic TiA1+ and ckit positive mast cells. These markers could help to predict at diagnosis the treatment response of patients with cHL and then to propose new targeted therapies to refractory patients. However it is likely insufficient to truly determine wether the immunohistochemical markers are independent of known clinical factors because of the small size sample. These results warrant further studies on a larger group of cHL by using micro-arrays technique which could also allow testing other biological molecules expressed in tumoral cells and in tumoral background.
